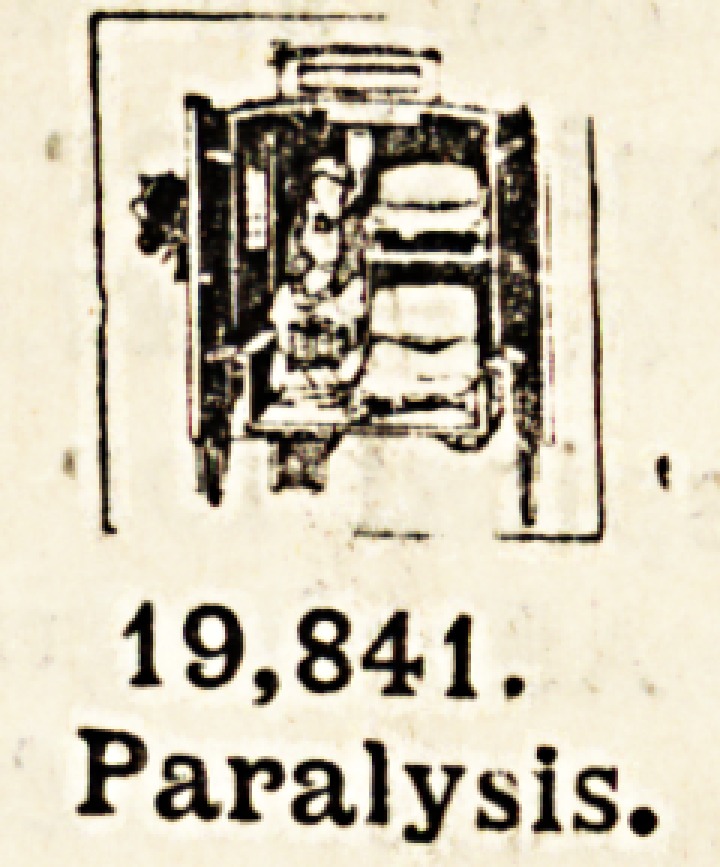# Special Hospital Sunday Supplement

**Published:** 1904-06-11

**Authors:** 


					The Hospital, Junk 11, 19C4,
Special Ibospital Sunba^ Supplement.
A Great Benefactor of our Hospitals.
MR. GEORGE HERRING.
We have great pleasure in presenting the hospital
*svorld to-day with an excellent portrait o? Mr. George
Herring who has already contributed .?53,000 to the
hospitals through the Hospital Sunday Fund. This
year, Mr. Herring, as on previous occasions, has
agreed to add one quarter to the amount collected in
?)lace3 of worship on Hospital Sunday, June 12, on
the sole condition that the amount shall be limited to
a collection not exceeding ,?100,000.
Mr. Herring is a gentleman who avoids every form
of publicity, and we therefore purposely refrain from
giving any biographical details of his life and history.
It is however of importance to the work in which
Mr. Herring takes so deep an interest that his
methods and views should be clearly understood.
On hospital matters Mr. Herring is entitled to speak
with authority, for he has devoted himself for many
years to the management and finance of one hospital,
the North-West London Hospital. This institution
ha3 developed and improved greatly under his direc-
tion. Mr. Herring has also established a large soup
kitchen in the poorest district of Kentish Town,
which has rendered great service to the poor, espe-
cially in the winter months.
In many directions, covering much of the field
of charity, often anonymously, but never without
fulness of knowledge and infinite kindness and care
Mr. Herring has given for years with a lavish hand!
His works are kno^n and greatly appreciated by many
leaders of public opinion, upon whose shoulders rests
The Hospital. June 11, 1904,
10 SPECIAL HOSPITAL SUNDAY SUPPLEMENT.
the largest share of the responsibility of providing
for the needs of the poorer classes throughout the
metropolis of the Empire. We have felt it essential
to say this much in regard to Mr. George Herring,
because we feel that his opinion upon the financial
requirements and possibilities of the hospital out-
look in London is of great practical importance and
entitled to carry great weight with it. What then
does Mr. Herring's experience and knowledge of
hospitals and charities generally teach 1
THE PROBLEM OF HOSPITAL REVENUE.
Two Main Sources of Support.
There are two kinds of donors who have proved
the mainstay of the philanthropic world for gene-
rations. Some men and women prefer to hoard their
wealth and
retain absolute *
control over it,
and to increase
it to the utmost
extent during
their lifetime,
and then to
bequeath it to
charity by will.
It would ill be-
come The Hos-
pital to under-
estimate the
value of the
dead hand, for,
as we show
in another
column, were
it not for the
revenues de-
rived from
legacies, and
the large
annual sum re-
ceived under
this head by
the hospitals,
it would be
impossible for
London to
maintain,
under existing
circumstances,
more than half
the hospital
beds which are
at present pro-
vided under
the voluntary
system.
Besides, as
we have often
shown in these
columns, most
of the success- i '
ful hospitals until about ten years ago were
cared for by a relatively few benevolent persons,
who not only gave of their substance, but who
directly and indirectly participated largely in the
administration of their affairs. It would not be
difficult to enumerate some hospitals which owe most
of their development and sustenance to a few
families and individuals who havo associated them-
selves with these institution, and have, therefore,
not unnaturally come to regard them as a sort of
freehold where their will must be law. Such a
result led to certain abuses, and on the whole tended
ultimately to
cripple both
the manage-
ment and de-
velopment of
particular
charities.
Nowadays
in London and
during the last
ten years espe-
cially, we are
glad to know
that the doc-
trine of per-
sonal service
has touched
the hearts of a
great number
of Londoners.,
and that
amongst men
of business in
the heart of
the City the
feeling has
grown thab
mere monetary
gifts, however
important in
themselves, do
not fulfil the
whole duty of
charity. Hence
it has come to
pass that not
one member,
but in some
cases every
member of
great firms of
merchants and
men of busi-
n e ss in the
City have ac-
, < ? cepted a seatr
on at least one hospital committee, with great
advantage to the , institution and not without
gain to themselves. , Mr.. George Herring i&i
an example of the truth of this statement, and
the fact that his association with one hospital'
for several years has led ' him to consider the
financial requirements' of' all the hospitals of
London, and then later to give tens of thousands of
The Hospital, June 11, 1904.
SPECIAL HOSPITAL SUNDAY SUPPLEMENT. H
pounds to these institutions through the Hospital
Sunday Fund. Mr. Herring evidently approves of
the Living Hand, and we agree with him. It is
clearly better for the individual and hotter far the
charities that a man or woman of wealth should give
liberally to the objects which interest them during
their lifetime, and should at the same time devote
a measure of their time and intelligence to the
management and expenditure of the money they may
be willing to provide from time to time.
The example of those who practise the doctrine
of the living hand must be fruitful in results upon
the age in which they live.
The Volume of Charity.
Men like Mr. Herring who are constantly thinking
out the problem underlying the voluntary support of
the hospitals of London realise that the volume of
charity in a particular year may be estimated with
reasonable certainty. Up to ten years ago the
amount contributed to hospitals year by year
was nearly stationary. One year it might vary
somewhat from another but the average was never
much exceeded in any period of twelve months.
The increase of 20 per cent, or so of the total
contributions to hospitals, comparing one decade with
another, when the figures were examined in conjunc-
tion with all the surrounding circumstances, con-
firmed this view. When considering a question like
this, it is too often forgotten that the material pro-
sperity of the citizens of London during the years
included in the calculation have an important bearing
upon the decision as to whether or no the volume of
charity is stationary or otherwise. This fact, Mr.
Herring has seen, and he, we believe, is striving
by his example, to induce thoughtful men of
means to look this question fairly in the face as a
matter of business, to assess the relative prosperity
of themselves and their neighbours and the relative
needs of the hospitals, and then more and more to
base the amount of their gifts to these charities,
upon the results shown by a consideration of all the
facts. In the last decade distinct progress has been
made in this direction, owing largely to the efforts
of the managers of the Metropolitan Hospital Sunday
Fund and to the establishment of King Edward's
Hospital Fund for London.
Hospital Rebuilding.
The volume of charity has a distinct bearing upon
hospital rebuilding. In intimating his gift to the
Sunday Fund. Mr. Herring states that he makes it
because the Hospital Sunday Fund is used only for
the maintenance of hospitals, and he fears that the
demands on the charitable for building, etc., may
deprive many deserving hospitals of part of their
present income, and so compel the closing of some of
the wards. He is therefore of opinion that the
King's Fund should not hoard the money entrusted
to it, but that its aim should be to devote to the
ho-pitals e^ery year all the money which the public
subscribe through its agency. No doubt the appeal
of St. Bartholomew's Hospital, which is asking the
public to subscribe ?500,000 without first formulat-
ing a definite scheme of rebuilding and supplying and
auditing an actuarial account setting forth the exact
present capital value of the invested funds and pro-
perty, has caused much misgiving in the mind3 of men
like Mr. George Herring who realise the whole posi-
tion and know what the average volume of charity, so
far as hospitals are concerned, in reality is. If the
great hospital with an enormous estate has to put
its hand into the hospital purse filled by the public,
the whole of the contents of which have before
been devoted with strict justice to supplying the
needs of the voluntary hospitals, Mr. Herring would
be justified, no doubt, in fearing that such a step may
deprive many deserving hospitals of part of their
present income. We believe that the British public
is more educated to-day on hospital questions than
ever before, and that in the result it will be found
that the appeal of St. Bartholomew's Hospital must
result in practical failure.
There must, after all, be a limit to the gifts to
hospitals by the public however much the aggregate
sum may represent. It follows that the larger the
aggregate sum contributed each year to hospitals the
greater will probably be the intelligence exhibited by
the donor3 in apportioning their gifts. If we are
correct, the managers of a great hospital possessed of
upwards of two millions of capital who have the
temerity to ask for ^500,000 more without making
out a case which is unanswerable and supplying a
scheme covering the whole expenditure which com-
mends itself to public approval, will find that the
response to their demands will in fact amount to
severe condemnation of their policy and methods.
The Outlook Ahead.
It is necessary to remember that the rebuilding of
old hospitals throughout London which have been
established upon the voluntary principle must be
faced and met every hundred years or so. Rebuild-
ing on a large scale is essential. In the past our
forebears and sires found the money to build the
hospitals originally and to reconstruct and rebuild
them as occasion required. To-day and during the
last ten years and upwards some millions of money
have been spent on rebuilding, etc., by the hospitals.
The amount still required will probably be found
not to exceed two millions in the whole, and when it
is forthcoming there should be no necessity for
any further great expenditure under this head for
many years to come. Of course scientific develop-
ment is much more rapid in these days, and it has
resulted in demands for hospital buildings of a type
so expensive as to be almost prohibitory. We incline
to the feeling that the present type of construction is
altogether too costly, and that a much simpler and
less permanent form of construction should be re-
sorted to. One reason why the cost is so great is
that the Building Acts in cities do not make allow-
ance for the requirements of special types of build-
ings, and we are of opinion that a great service
would be rendered to the hospitals and the public if
a conference of architects and designers of hospitals
were to be held to consider this aspect of the
hospital problem to day. Then, again, it is en-
couraging to notice that there is a steadily in-
creasing growth in the numbers of those who are
beginning to realise that in the days of health they
owe something to the cause of suffering and sick-
ness, for so the number of givers to hospitals is bound
to increase. Public opinion, too, is ripening in the
direction of radical reform in the administration of
the hospitals in our great cities. At the present
The Hospital, June 11. 1904.
12 SPECIAL HOSPITAL SUNDAY SUPPLEMENT.
rate the medical profession will place themselves
and their brethren in the bankruptcy court, where
they may speedily land the voluntary hospitals too,
unless the present craze for granting free medical
relief to everybody who applies for it is speedily
cured. We have only space to indicate these
few aspects of the outlook ahead, but we commend
them to the careful consideration of all who support,
as well as of those who administer the voluntary
hospitals of this country.
A Word to Liying Londoners.
Wats and Means.
Year by year in our Special Hospital Sunday
Supplements we have given statistics to show the
proportion of the money given for the care of the
sick in London by (1) the living, namely, the pre-
sent inhabitants of the Metropolis, (2) by deceased
benefactors and (3) by the patients themselves. "We
have pointed out how inadequate is the sum con-
tributed by the living to insure the good work which
is done by our hospitals being maintained. The latest
complete returns are not calculated to make us particu-
larly satisfied with ourselves. We find that in this
year (1902), including St. Bartholomew's Hospital, the
voluntary contributions amounted to 8s. 5d. in the
pound, which, added to the Is. 5d. received from the
patients themselves, brings the amount given by the
living up to 9s. lOd. in the pound, a slight increase
on the amount received in 1901. When, therefore,
Londoners are inclined to feel proud of their exceed-
ing liberality to the hospitals they deceive themselves.
That is a mean and contemptible spirit which takes
credit for virtue it does not possess. If no such
reproach is to rest on London this year it is im-
perative that all classes should give more liberally
to the Hospital Sunday Fund and persuade others
to follow their example.
The Income Available for the Work Done.
In the year 1902 hospital treatment was provided
for about two million and one hundred and ten
thousand persons, exclusive of the patients treated
at the hospitals of the Metropolitan Asylums Board,
and the total ordinary income and legacies received
by the London voluntary hospitals and dispensaries
for this purpose was ?1,201,385, which was derived
from the following sources :?
Charitable or voluntary contri-1 0(0 An
butions ... ... ...J 10,248 or 42 per cent.
Income from invested property 269,476 ? 23 ?
Legacies  839,091 ? 28 ?
Patients' payments   82,570 ? 7 ?
. So far as the above figures refer to St. Bar-
tholomew's Hospital they have been confined to
that portion of the revenue which is applicable to
hospital purposes.
How the Money is Provided.
The mere statement that such and such an amount
of money has been raised from one source or another
does not, as a rule, greatly impress people. It is
rather the difficulties which attend the raising of it,
and the consideration of the sources from which it
comes, that enable us to judge whether those who
are in a position to give are doing their duty. It is
therefore of the first importance that everyone should!
thoroughly understand where the money comes from
to pay the cost of the relief given by the hospitals
to the inhabitants of London, and to make this as
clear as possible we have prepared diagrams, each
representing a hand and a coin, which have been
drawn to scale to show the proportion of every
sovereign contributed by the living, i.e., those who
receive the benefits, and by deceased benefactors,
many of whom have not only left their money to
enable the good work to be carried on, but were also
during their lives active workers for the hospitals.
With a view to clearness the diagrams have been
drawn to represent the proportion of every sovereign
given in 1902 by (a) the dead, (b) the living, and
HOSPITALS AND THEIR SPECIAL NEEDS.
Bolingbroke Hospital, Wandsworth Common,
S.W. ??20,000 lor new buildings and ?700 to pay
outstanding accounts.
British Home and Hospital for Incurables,
Streatham, S.W.?Deficit for 1903, ?G,500.
British Lying-in Hospital, Endell Street, W.C.?
?595 to make good last year's deficit; the repayment
of ?5,000 raised to build nurses' home.
Cancer Hospital (Free), Fulham Road, S.W.? ?6,000
to provide fitting accommodation for nurses in their
trying work at this hospital, and to equip a patho-
logical and electrical department for cancer research.
Central London Ophthalmic Hospital, Gray's Inn
Road, W.C.?Funds required to rebuild, as urged by
King Edward's Hospital Fund. Present accommodation
unsuitable. No endowment.
Chelsea Hospital for Women.?Increased donations
and annual subscriptions to provide for continuous
growth in the relief afforded.
City of London Lying-in Hospital, City Road, E.C.
??25,000 for the rebuilding of the^old portion of the
hospital, which has become imperative. ?1,000 to meet
extra expense of temporary in-patients' department.
The Dead Hand
gave
THE DEAD HAND GIVES 10S. 2d. OUT OF EVERY ?1
RECEIVED BY THE HOSPITALS.
The Hospital, June 11, 1904.
SPECIAL HOSPITAL SUNDAY SUPPLEMENT. 13
(c) the patients themselves. The black hand and the
coin held by it represent the contributions from those
dead ; the white hand represents the charitable
contributions from the living, i.e., ourselves, the
living Londoners ; and the smallest coin indicates
the amount received from patients' payments.
Of every sovereign received 10s. 2d., or more than
half, is derived from legacies and the interest on
9ifts from deceased benefactors, which have been
invested in approved securities ) 8s. 5d. out of every
Sovereign has been given in charity by the present
inhabitant of London, that is, the living, for the
benefit of whose generation the hospitals exist; and
Is. 5d. of every sovereign has been contributed by
those who have been actually under treatment in the
hospitals. Let us study these figures carefully, and
Understand why they are so unsatisfactory, why they
do not inspire us with confidence. It will be seen
that a considerable portion of the 10s. 2d. given by
the dead hand is derived from the legacies received
during the year?to be exact, 5s. 7d. of it?and this
source of income mast always be fluctuating. As
compared with 1901 there is an increase of nearly
?30,000 in the amount received from legacies, but
in 1896 there was a sudden drop of ?40,000 and in
1900 one of over ?70,000. So the sum given by the
dead hand may fail us sometimes, and this should be
recognised by the living. A fact like this brings us
face to face with the certainty that, unless such de-
ficiency is made up by the increased gifts of the
living, the great work of our London hospitals must
be seriously crippled.
Now let us see how the gifts of the charitable
compare this year with last. The actual amount
received is larger than in either 1900 or 1901, and
represents 8s. 5d. in the sovereign as against 8s. 2d.
and 8s. 7d. in these two years, but it must not be
forgotten that even though it has increased slightly,
were it not for the legacies received each year
the income of the voluntary hospitals would be
only equivalent to about 15s. or 16s, for every
sovereign which the managers of those institutions
have to expend on the service of the sick and
suffering in the metropolis. The amount received
from the patients themselves is Is. 5d. in the ?1, as
against Is. 5d. in 1901 and Is. 7d. in 1900.
The Meaning of the Diagrams.
We most earnestly ask all classes in London to
spend a few moments in considering the facts dis-
closed by these figures. While the people of London
make use of the hospitals in ever-increasing numbers,
the figures prove that they are not ready to make an
adequate return for the benefits received, but are
content to trust to the dead hand to make up any
deficiency. Unless this lamentable lassitude in
our charity gives place to more honest endeavour,
the ultimate result is not far to seek. In London
to-day there are some hospitals unable to take in
the patients asking for admission, sometimes be-
cause the hospital is not large enough to cope
with the district it serves ; occasionally because
beds are closed for want of funds. Look at this
fact from whatever point we will, it is one to
make us ashamed. We boast of being citizens of this
rich and powerful centre of the greatest Empire the
world has known, yet we allow ourselves to
remain in imminent danger of the reproach that in
spite of our riches, in spite of our power, we grudge
the help our suffering and less prosperous brethren
need. After all, it is only a very small sacrifice on
the part of each individual that is required, and to
plead inability is in ninety-nine cases out of a hundred
absolute dishonesty. You cannot afford to give a
shilling or two to the hospitals, but you never think
twice about giving half-a-crown, probably many
times a year, for a place in the pit of a theatre. We
can all do something. It is our duty ; it should be
our pleasure. Whether we like it or not, the care
of the sick is entrusted to us, and who can be
callous enough to neglect such a trust 1
hospitals and their special needs.
East London Hospital for Children.?Accumulated
deficit up to date, ?2,683. Balance of ^6,000 required
to complete erection of wards necessary for whooping
cough and other isolation cases. Badly in need of
more accommodation for patients and nurses.
Grosvenor Hospital for Women and Children,
Vincent Square, Westminster, S.W.?Additional
support to throw open 12 more beds and to provide
suitable accommodation for nurses.
Gordon Hospital, Vauxhall Bridge Road, S.W.?
?5,945 to remove burden of debt. ?6,950 to complete
the hospital.
Guy's Hospital.??77,000 to discharge balance due for
renovation and building. Additional ?15,000 a year to
work the hospital at its required strength.
Great Northern Central Hospital, Holloway Road,
N.??2,500 required for necessary improvements, aLd
?1,898 to remove deficit for 1903.
Hospital and Home for Incurable Children, 2 Maida
Vale, W. ?Present accommodation structurally de-
ficient and insanitary. Donations greatly needed to pay
for suitable premises ?which have been secured at Swiss
Cottage.
(iContinued on page 16.)
The Laving
gave
8s. 5d. in
the ?1.
TBE living, i.e., THE PRESENT INHABITANTS, GIVE 8S. 5D.
OF EACH ?1 RECEIVED BY THE HOSPITALS.
Patients' Payments
yield
/ ?5*? ? ai
Is. 5d. in
the ?1.
patients' payments supply Is. 5d. of EACH ?1 RECEIVED
BY THE VOLUNTARY HOSPITALS.
The Hospital, Jdne 11, 190*'
14 SPECIAL HOSPITAL SUNDAY SUPPLEMENT.
Oyer Two Million Sufferers Helped by the Hospitals.
A SINGLE YEAR'S ROLL-CALL OF THE SICK.
How many patients are annually treated in the
London Hospitals 1 The cases which we have sorted 1
out under the various headings comprise those treated
at the voluntary hospitals and dispensaries of London
?together with the endowed hospitals of St. Bartholo-
mew's, Guy's, and St. Thomas's?and the hospitals
of the Metropolitan Asylums Board. Altogether
they reach the immense total of two million two
hundred and eighty-nine thousand Jive hundred and
seventy-eight patients.
Patients Suffering from Surgical Diseases.?Of
the whole number of patients received by the hospitals,
nine hundred and fifteen thousand nine hundred and
twenty-seven required surgical treatment. Let us
clearly understand what is meant by " surgical
diseases." They include not only all accidents such
as broken bones, fractured skulls, mangled limbs,
and all manner of displacements and crushings of
sensitive parts and organs, but also abscesses, ulcera-
tions, cancers, and tumours of all kinds ; in short all
those injuries which may be produced by accident or
pathological process, and which may be dealt with
?either by hand or instrument. That such a vast
number of persons in London suffer from one or
more of the injuries thus briefly summarised is not
-easy to realise. They form a very large army indeed,
these 915,927 sufferers.
Patients Suffering from Medical Diseases.?Seven
1hundred and fifty-four thousand eight hundred and
forty-nine persons received medical treatment. By
medical diseases is meant those diseases which are
situated either as to their source and origin or in their
entirety in one or the other of the three great cavities
of the body. They include rheumatic fever, pneumonia,
pleurisy, bronchitis, diseases of the stomach, bowels,
liver, kidney, bladder, and pancreas, every kind of heart
disease, many forms of brain injury, dyspepsia, consti-
pation, most nervous diseases, and other ailments,
many of them serious and many of them dangerous to
life, or at least to the useful existence of the in-
dividual. Remember that most of these diseases are
out of sight, that the diagnosis of their nature and
extent, and the successful treatment of them, is
dependent on the doctor's scientific knowledge, and
then try and realise that in the hospitals of London
over 750,000 persons received treatment at the hands
of the foremost physicians of the day, free of cost to
the patients themselves. Surely when the hospitals
plead for help to us who are in health our answer
should be a generous one. Good health is a free gift,
and since we have received so lavishly we ought cer-
tainly, as a thankoffering, to give liberally.
Jfatients Treated at Special Hospitals for Children.?Included in the 575,000
children mentioned at the commencement of this article are one hundred and forty-
nine thousand two hundred and thirty-four children who were sent from homes
where they could not be properly attended to for treatment in the special hospitals
for the little ones. To see the small face pinched and pain-marked, instead of sunny
and dimpled with smiles, the tiny limbs inert and still which should be full of joyous
movement and new life, and to hear a low wail of agony from lips which were
formed for childish prattle, must pierce the heart of every man and woman worthy
the name. Parents who are happy in the knowledge of the noisy nursery at home,
whose very lives echo with the pleasant pattering of little feet, cannot withhold their
hands. When the children's hospitals appeal for funds to carry on the work of
restoring to health and strength the little ones of this great city, the children who
will one day take our place as the workers in this land of ours, let the children
who are strong and well give to those who are ill and weak.
915,927.
Surgical Patients.
754,849.
Medical Patients.
149,234.
Children.
Thk Hospital, June 11, 1904.
SPECIAL HOSPITAL SUNDAY SUPPLEMENT. 15
THE ROLL-CALL OF THE SICK .?Continued.
Patients Suffering from Eye Affections. ? One hundred and Jorty- seven
thousand seven hundred and thirty-six persons were treated in the special de-
partments of the general hospitals or by the ophthalmic hospitals of London. It
ls certain that very many of these cases must have entailed terrible suffering, and
many doubtless would have terminated in total loss of sight but for the skilful
treatment they have received at the hospitals. Who can say how many have
been saved from becoming practically helpless in the world 1
Diseases of Women and Motherhood.?One hundred and three thousand seven
hundred and fifty-nine women were treated at the metropolitan voluntary hospitals
for those diseases which are peculiar to their sex. Here it is not only our
sympathy which is appealed to, but our patriotism as well. Here there is an actual
demand for the payment of a debt we most justly owe. The very heart and strength
of the nation lies in the home life, and the soul of the home life is the woman?the
mother. The majority of us are to-day what we are because of the influences brought
to bear upon us in the home.
Patients Suffering from Diseases of the Ear, Nose, and Throat.?At the special
hospitals or special departments devoted to these diseases sixty eight thousand two
hundred and thirty-two were treated. The affections and diseases of these organs,
which are intimately connected, involve temporary and often permanent impairment
of hearing, swallowing, and breathing. These functions are performed with so little effort
on our part that, unless experience has taught us, it is difficult to understand what it
would mean to us if we suddenly had to suffer from one or other of these affections.
Patients Suffering from Diseases of the Skin?During the year fifty thousand six
hundred and fifty-four persons were treated for skin diseases in London. It is, perhaps, more
difficult to bring home to people the claim which sufferers from skin diseases have upon
their sympathy than it is in any of the other classes of disease which we are considering.
There is not, as a rule, the pain, nor the danger to life, nor even such risk of permanent
disablement as is the case with many of the others j but let us remember what the result
would be were there no hospitals for the sufferers to go to.
Patients Suffering from Consumption.?Forty-eight thousand two hundred and forty-
two patients suffering from phthisis or consumption were treated at the consumption
hospitals of London during the year. The very word consumption makes us afraid. There
are few of us who have not seen something of its ravages, of its cruelty. Truly may con-
sumption be called the curse of our climate. It respects neither persons nor estate, neither
rich nor poor, the old or the young.
Patients Suffering from Fever.?The number of persons who suffered from various
forms of fever during the year was 31,104. This figure is however a misleading one,
because the term fever includes much besides the class of fevers which are usually removed
toThese hosSs Mclsles for instance, prevails in London to sneh an extent that more
deaths occur from it than from scarlet fever. The excellent service rendered by the London
Fever Hospital entitles it to the gratitude of all householders. , .
Patients Suffering from Paralysis and Diseases of the Nerves.?Nineteen thousand
eight hundred and forty-one stricken with paralysis and similar diseases of the brain received
treatment at the hospitals devoted to these maladies. To workers busy with hand and brain
these sufferers must particularly appeal. It is impossible to disassociate nervous breakdown
from the toil and hurry of existence, especially in a vast centre like London. It is appalling
to think that at any moment any one of us may be struck down suddenly, perhaps without the
slightest warning. No disease is more sudden than paralysis, surely none more pitiful.
So this great army of sufferers, numbering over two and a quarter millions, claim our
sympathy and our help year by year. To the strong, to those in health who are able to
provide for those dependent upon them, to those who know what ill-health means, who
have suffered from disease of one kind or another, and who, either in the hospital or
under the skill and care of the doctors and nurses trained in the hospital, have been
restored to health and usefulness, we confidently appeal on behalf of the London hospitals.
Sufferers needing Surgleal Aid . . . 915,927
? Sufferers needing Medical Care . . . 754,849
Children  149,234
Sufferers from Eye Troubles . . . 147,736
Diseases of Women  103,759
Diseases of the Ear, Nose, and Throat . 68,232
THE ROLL-CALL OF THE SICK.
Sufferers from Skin Diseases. . . . 50,654
Consumptives  48,242
Fever Patients 31,104
Paralysis   19,841'
Total  2,289,678
147,736.
Eye.
103,759.
Women.
68,232.
Ear and Throat.
50,654.
Skin.
48,242.
Consumption.
31,104..
Fever.
19,841.
Paralysis.
? The Hospital, June 11, 1904;
16 SPECIAL HOSPITAL SUNDAY SUPPLEMENT.
Some of the Debt We Owe to Medicine.
There are but few individuals who do not at
some time or other in the course of their lives feel
a debt of gratitude to the medical practitioner.
When health and activity pulsate in every vein
the doctor, it is true, is hardly included in our plans
and calculations. But when pain and disease make
themselves recognised the cry for assistance and
relief is one of the most urgent of human claims.
And when by medical skill and attention pain is
assuaged and health restored he is of curious mould
who does not feel the gratitude that is due to the
art and the man who have made him ever their
debtor. As this is for most people a direct
personal experience it is not necessary to empha-
sise the indebtedness to medical science which
is established by the relationship between the
individual patient and the individual physician.
Life and experience proclaim it, and all thoughtful
persons gratefully and heartily recognise it. But the
debt of the community to medical science is far from
being exhausted by the recognition of individual
assistance and relief. Indeed, when considered from
a broader point of view and studied as a contribution
to human welfare, the debt of mankind to medicine
is seen to assume an even more formidable total.
This is well illustrated by the rule and practice
according to which any method of treatment intro-
duced or discovered by any member of the medical
profession is placed without reserve at the disposal
of the entire profession, and thus becomes available
for sufferers the wide world over. Again and a gain this
has meant a large pecuniary sacrifice by the individual
practitioner, and at the same time has carried an
incalculable boon to suffering humanity. Take a single
instance. Some 30 years ago the late Dr. T. J. Mac-
lagan made a careful investigation into the causes and
pathology of fevers, and was led by a series of steps
to the discovery of the salicyl compounds as remedies
in the treatment of rheumatism. What has this
meant for the human race 1 It ha3 meant that the
hundreds of thousands of men, women and children
who since the discovery have suffered from rheu-
matism, especially in its more acute forms, have been
promptly relieved of their pain, have been more
rapidly restored to health, and have been saved from
the risk of severe and dangerous complications.
Think of the enormous money value this signifies in
the family and national life. It is not only the
individual sufferer who receives benefit; it is, in
addition, the community at large, whose total work-
ing capacity is multiplied and enlarged. Now this
discovery and all that it means were given to the
nation as a free gift by the medical profession
in the person of Dr. Maclagan. Suppose he
had chosen to pursue the opposite course and had
kept his discovery a secret, retaining the use
of the salicyl compounds for those who placed
themselves under his treatment. Unquestionably
he would in the course of a very few years have
accumulated an enormous fortune. Patients and fees
would have poured in upon him. But the great
proportion of sufferers from rheumatic fever would
have remained without relief. Fortunately, in ac-
cordance with the universal practice of his profession,
Dr. Maclagan preferred the welfare of humanity to
his own personal aggrandisement. He added his
new knowledge to the common stock, and thus
placed it within the reach of every patient. Probably
but few members of the general public have ever
heard his name, and no signal honour or reward ever
came to him. Yet he gave to humanity a great
boon, and it is to his everlasting credit and to
the credit of the profession which he represented,
that so far from his conduct exciting surprise it
was taken as the natural and proper thing to do.
Every piece of new knowledge, every suggested im-
provement in the treatment of disease, instead of
being reserved and utilised for private gain, is given
freely and without stint for the benefit of the entire
human race. In this way is accumulated an advan-
tage for which all the world is debtor. But it is
not alone to the medical profession that the debt is
owing. For many reasons the hospitals also have a
direct claim for gratitude from every individual and
from the nation as a whole for the great advances
which have been made in nearly every branch of
medicine and surgery. Indeed, it is not too much
to say that to the experience gained in the wards of
our hospitals, and to the large mass of material for
clinical observation that is provided in these institu-
tions many of the most far-reaching of recent dis-
coveries are partly due. Everyone knows and
honours the name of Lord Lister, and the work
he has accomplished. His system of antiseptic
surgery is practised all over the civilised world,
and on a moderate estimate saves 50,000 lives and
50,000 limbs every year. Can anyone calculate
or express how much mankind is the debtor to
this distinguished member of the medical pro-
fession 1 And yet it should be remembered that this
great work could hardly have been accomplished
but for the existence of hospitals. Only with
HOSPITALS AND THEIR SPECIAL NEEDS.
Hospital for Sick Children.?More income. ?1,000 will
endow a bed in perpetuity. A large sum required to
erect an out-patients' department.
Hospital for Women, Soho.?The extinction of the
debt of ?7,500.
Hospital for Gentlewomen, 90 Harley Street, W.?
Affords gratuitous medical treatment. Requires in-
crease of ?800 annually in voluntary support and ?223
to meet deficit for 1903.
Hospital of St. John and St. Elizabeth, Grove End
Road, N.W.??1,238 to remove last year's deficit.
Additional support to re-open ward with 17 beds which
cannot be maintained with present income.
Italian Hospital.?Additional income much needed to
provide relief for a large proportion of English as well
as Italian patients.
King's College Hospital.?Assistance to meet current
expenses, pending the removal of hospital to South
London.
London Fever Hospital.?About ?10,000 to defray cost
of reconstruction of very old buildings.
London Hospital, E.??96,G95, being the sum estimated
as necessary to complete scheme of structural exten-
sions and improvements. To meet annual expenditure
of ?90,000, only ?17,000 can be relied upon in income.
The Hospital, June 11, 1904.
SPECIAL HOSPITAL SUNDAY SUPPLEMENT. 17
a considerable number of patients collected in cir-
cumstances which permitted exact and continuous
observations could it have been possible to have traced
the cause of suppuration in wounds, and to
have devised a method which has made the art
of surgery so safe and so successful. The debt to
hospitals therefore falls not only on those who are
treated there as patients. It covers all who gain
restored health by the help of modern medicine and
surgery, for it is in these institutions to a very large
extent that there has been obtained that more com-
plete knowledge of disease which makes successful
treatment possible. Another development is the
treatment of certain diseases by anti-toxins. This
is perhaps best known in connection with diphtheria.
The value of this method is seen by comparing the
statistics of the hospitals of the Metropolitan
Asylums Board in 1890 with the figures for 1900.
In the former year the deaths among cases of
diphtheria were 33*5 per cent., whilst in 1900
the percentage mortality had fallen to 12'01, and
parallel figures could be quoted from all parts
of the world where this treatment has been adopted.
What is true of diphtheria will almost certainly ere
long be shown to be true of a number of other
diseases, such as tetanus, plague, and typhoid fever.
Expert bacteriologists are investigating the minute
organisms which cause these and similar diseases,
and it may be taken as certain that when the cause
of any disease is fully understood it will not be long
before means are devised for its prevention or cure.
This is well seen in the case of malaria. So long as
ignorance prevailed in reference to the causes of this
disease little or no progress was made in treatment.
But when a few years ago malaria was shown to be
due to the presence of a parasite in the blood which
is conveyed to man by the bite of the mosquito,
the position was completely changed. And now there
is every reason to expect that many tropical districts
which are now so dangerous to the white man will
be freed from the disease which constitutes their
curse. Such a result makes a special appeal to
men of British blood who have active interests in
so many parts of the earth's surface. If only certain
British possessions can be freed from malaria their
economic value will be enormously increased, and the
work which is being prosecuted in this direction has
every promise of a large success. Here again medi-
cine confers its boons not merely on actual or poten-
tial sufferers but on the entire body of the nation.
Another item in the total of debt due to medical
science is found in the elaborate scheme of organisa-
tion by which the community is protected against
the outbreak of epidemic disease. It is only by a
vigilance that never relaxes that this end is secured.
Here and there in spite of these efforts a limited out-
break occasionally occurs. But in countries where
medical and sanitary precautions are adopted the
people dwell in safety and are practically free from
the terrible epidemics that in former days swept away
in the course of a few weeks a large proportion of
the population.
What we owe to medicine cannot indeed be ex-
pressed in terms of pounds, shillings and pence, but
none the less, healthy as well as sick, the individual
citizen, the municipality, and the nation are all its
debtors both for the good it accomplishes and the
evil it prevents. Many of its greatest triumphs have
meant great personal sacrifices for the cause of
humanity. Its work is concerned both with the
pathos of individual suffering and the achievement
of national efficiency.
Helpers on Hospital Sunday.
The Preachers.
There are three classes of helpers who, if they
will only address themselves to the work with that
touch of enthusiasm which sets human hearts on fire,
can make Hospital Sunday, 1904, a record anni-
versary. The preachers have a splendid opportunity.
If there are " sermons in stones," there should cer-
tainly be in the needs of the sick and suffering material
for the most brilliant and moving discourses. It should
not at any time be a complaint of the clergy that they
cannot find a theme which affords scope for the exer-
cise of eloquence; but even the man who is most des-
titute of oratorical gifts may discover inspiration in
such a topic as that which is ready to his hand next
Sunday. There is no occasion for him to consider
the conventional divisions which seem so often to be
regarded as essential. His exordium, his points, and
his peroration are all provided for him. It might
refresh his memory?for ministers of all denomina-
tions are, we presume, familiar with the interior of
some of our great charitable institutions?if he could
spend an hour in the wards of a hospital. But
HOSPITALS AND THEIR SPECIAL NEEDS.
London Lock Hospital, Harrow Road, W.?Money
to carry out long delayed renovations, Sadly in need
of annual subscriptions.
Metropolitan Hospital.?Entirely dependent on casual
and uncertain income.
Middlesex Hospital, W.? Money to replace large capital
outlay on extensive structural improvements and
extensions.
Miller Hospital.??5,000 to complete necessary extensions
and improvements.
Mount Vernon Hospital for Consumption (Open-air
Treatment), Hampstead, N.W.?Debt of ?15,000
on building account must be repaid before more beds
are opened, and there are 20 applicants for every bed at
present available. ?1,500 to remove deficit for 1903,
arid support for current expenses.
National Hospital for Paralysed and Epileptic,
Queen's Square, W.C.??6,000 required to repay
loan for current requirements; ?500 towards cost of
new operating theatre.
National Sanatorium for Consumption, Bourne-
mouth.??500 more income to provide for increased
number of patients. ?1,000 to complete payment for
necessary renovations. ?305 to pay outstanding
accounts.
New Hospital for Women, Euston Road.?Deficit
for 1903, ?973. Another ward required to relieve
pressure upon accommodation.
Tee Hospital, Junk 11, 1904.
18 SPECIAL HOSPITAL SUNDAY SUPPLEMENT.
without aDy effort of imagination he can people his
mind with the scenes which will enable him
to describe in true and graphic language the manner
in which the commands of his Master are carried
out inside its walls. If he can demonstrate thip,
and give practical proof of the fact that the bodies
of men, and women, and children are being cared for
as they were cared for by Him who went about
doing good, his appeal must compel attention on the
part of a congregation consisting of persons
professing to be Christians. If there are
preachers who like to be considered up to date,
they might cite the growing tendency for ren-
dering service which ought to be voluntary com-
pulsory, as a strong reason for assisting the hospitals.
For all these centuries the English nation has
depended upon the patriotism of her sons to rally to
her defence in the event of war, but if the report of
a Royal Commission which has recently been issued,
is ever carried into effect, our youths will have to be
trained as soldiers whether they like it or not.
There may be arguments in favour of conscription,
but the substitution of State-supported for voluntary
hospitals would deprive this country of the finest
evidence which can b9 provided of British
generosity in the most beneficent of causes. With
the disappearance of those golden letters, " Supported
by voluntary contributions," from the vestibules of
our hospitals would disappear also the motive for
obedience to one of the most solemn injunctions of
the founder of the Christian faith. Yet if the im-
perative necessities of the hospitals are not met by
the freewill offerings of the charitable, the day
cannot be far distant when the advocates of com-
pulsory service, even in respect to the sick and
suffering, will present an overwhelming case, and
one of the springs by which the noblest instincts of
mankind are nourished will be in danger of being
dried up.
The Press.
It is a moot question whether the power of the
pulpit or that of the press is the greater. Bat it is
beyond doubt that the press can, if it chooses, be a
magnificent agency in the interests of a deserving
cause, and we do not conceal our opinion that the
London press might do far more than it has yet done
for the London hospitals. In the London season,
with Parliament sitting and a terrible war in progress,
space is, of course, extremely valuable, and it would
not be fair to ask for unreasonable encroachments upon
it. But our contemporaries, who have rendered such
great help in the past, might still further increase
the indebtedness of suffering humanity to them if
his year they would tell their readers, even to the
extent of wearisome reiteration, of the approach of
Hospital Sunday. Forgetful minds require to be
jogged, and the occupied Co bi reminded of an annual
duty. The publication of a list of preachers at the
churches and chapels on Hospital Sunday in the
daily papers, with an intimation that the offertories
will be for one object, is extremely useful; and if
this were supplemented, not only by announcements
of the amounts collected, but also by a number
of brief summaries of the most noteworthy
sermons rather than by long reports of a few,
the interest excited would be the wider. It is,
perhaps, most of all by exciting among different
congregations the spirit of wholesome competition
that the press can be the means of substantially
augmenting the total sum collected during the day.
The Public.
But, after all, the lion's share of obligation in the
matter falls upon the public. In vain will the
preachers exhaust their stories of pathos in urging the
claims of Hospital Sunday, and the press their chances
of making known the event, if the public turn a deaf
ear to the one and remain indifferent to the other.
Hospital Sunday should at least be a day on which
every church or chapel goer attends a place of worship
and does something to swell the collection. Cyclists and
motorists who pass by the church or chapel doors
in search of pleasure or fresh air, can, at any rate,
satisfy their consciences, so far as their debt to the
hospitals is concerned, by sending the clergyman of
the parish, or the minister of the chapel, the shilling,
half-crown, or sovereign which they can spare. The
aim of every particular parish, or congregation, not
excluding the children, who cannot too soon be
taught the importance of helping the sick who
cannot help themselves, should be to bear wit-
ness, in a manner that cannot be mistaken, to
the reality of their charity. If the individual man,
the individual woman, would remember that upon
their action the ultimate result depends, instead
of leaving their burden to others to discharge, the
Council of the Hospital Sunday Fund would have
the pleasure of receiving from the churches, nob
?50,000, but ?200,000. It is the contribution of
the many that is wanted?the pence of the grateful
who have benefited directly from the hospitals ; the
silver of those who have never experienced the
necessity of seeking refuge in them ; the gold of
those who have been tended in their own homes by
the best physicians and the most highly-trained
nurses. Given preachers burning with zeal, a press
concerned to rise to the fulfilment of its highest
mission, and a public determined to recognise their
duty to their neighbour, no forecast of the issue of
the appeal on Sunday could be too sanguine.
HOSPITALS AND THEIR SPECIAL NEEDS.
North Eastern Hospital for Children.?Deficit for
1903 is ?869. ?9,000 urgently required to pay debt for
enlarging hospital, and ?5,000 for general maintenance.
North-west London Hospital, Kentish Town Road,
N.W.??2,000 to make good last year's defisiency.
The treasurer, Mr. George Herring, promises to add one-
third of all contribution?.
Paddington Green Children's Hospital, W.?Owes its
bankers ?2,600; reliable income short of necessary
expenditure by ?3,400; contributions requires ior up-
keep of convalescent home at Slough.
Poplar Hospital fop Accidents, Poplap, E. -The
working men give ?600 a year, and the wealthy are
asked to give ?8,000 to defray the annual expenses.
Queen Charlotte's Hospital, Marylebone Road,
N.W.?Increased demands render further provision
necessary, but to effect this ?4,000 is required.
The Hospital, June 11. 1904.
SPECIAL HOSPITAL SUNDAY SUPPLEMENT. 19
Metropolitan Hospital Sunday Fund.
The Metropolitan Hospital Sunday Fund has now-
been established for a little over thirty years, and on
looking back along this period and taking, so to
speak, a bird's-eye view of its history, one has the
satisfaction of observing an unbroken career of
prosperity and increase. In the year 1873, during
the first twelve months of the Fund's existence, the
total sum collected for the hospitals was ?27,700 j
in 1903 the receipts were ?64,975 ; while in the
period under review the contributing congregations
increased in number from 1,072 to 1,987. In other
words, the efforts put forth by those men who
realised the need of the hospitals and who insti-
Table showing the Results of Collections since the Covimence-
vient of the hind.
Contri-
buting
con-
grega-
tions
Amounts re-
ceived from
congregations
Legacies,
special dona-
tions and
dividends
rotal receipts
1,072
1,217
1,120
1,089
1,165
1,171
1,176
1 225
1,268
1,337
1,414
1,522
1,597
1,595
1,633
1,669
1,655
1,712
1,711
1,741
1,772
1,799
1,807
1,811
1,793
1,785
1.797
1,804
1,860
1,925
1 987
? s. d.
25.855 13 0
28,431 7 6
24 971 16 3
25,703 7 2
25,135 18 4
23,705 10 3
25,380 4 4
28.856 17 4
30,218 7 10
32,039 13 6
31,954 7 2
32,918 18 7
31,764 17 1
35,581 18 9
36,476 17 6
37,299 7 1
38,272 4 7
38,823 2 1
36,310 17 6
37,082 15 2
35,638 13 3
35,962 5 3
38,370 11 7
40 501 11 9
37,396 2 2
36 532 18 11
38,194 0 11
35 856 8 11
36,388 6 4
46 362 8 3
49,209 2 11
? s. d.
1,844 15 1
1,505 10 4
1,424 5 9
1,339 4 2
947 0 9
1,199 9 3
1,120 19 9
1,567 1 6
1,638 6 7
2,106 8 11
1,980 19 1
6,410 17 11
2,555 11 4
4,817 8 10
4,130 10 2
3,080 2 5
3,482 8 4
3,991 14 8
9,019 15 11
4,429 7 6
3,652 6 8
7,717 6 5
21,990 12 7
5,533 11 3
3,607 10 11
3,864 3 11
15,310 10 2
16,137 5 7
18,343 12 11
16,307 4 4
15,766 10 6
? s. d.
27,700 8 1
29,936 17 10
26.396 2 0
27,042 11 4
26,082 19 1
24,904 19 6
26,501 4 1
30,423 18 10
31,856 14 5
34,146 2 5
33,935 6 3
39.329 16 6
34,320 8 5
40,399 7 7
40,607 7 8
40,379 9 6
41,744 12 11
42,814 16 9
45.330 13 5
41,512 2 8
39,290 19 11
43,679 11 8
60,361 4 2
46,035 3 0
41,003 13 1
40.397 2 10
53,504 11 1
51,993 14 6
54,731 19 3
62,669 12 7
64,975 13 5
tuted and carried on the work of the Metropolitan
Hospital Fund with the special object of meeting
this need, have resulted in more than doubling, in
the short space of 30 years, the large sum received
in the initial year. The above table shows at a
glance the sustained and rapid development of the
work, the increase of contributing congregations, and
of the sums annually received. Even more striking
than these are the figures which we give below show-
ing for purposes of comparison the results of three
successive decades of the work :?
Decades.
1873-1882
1883-1892
1893-1902
Amounts re-
ceived from
congregations.
Legacies,
special dona-
tions and
dividends.
? s. d.
270,298 15 6
356,485 5 6
382 203 7 4
? s. d.
14,693 2 1
43,898 16 2
112,464 4 9
Total receipts.
? s. d.
284,991 17 7
400,374 1 8
493,667 12 1
It will be seen from the former table that no less
a sum than ?1,244,009 4s. 9d. has already been
collected for the hospitals and medical charities of
London through the agency of the Metropolitan
Hospital Sunday Fund in the 31 years of its exist-
ence at an average annual cost of but 3 7 per cent.
This is an achievement of which all concerned may
well be proud, the ministers of religion, the con-
tributing public, and the active members of the Fund
alike. And even greater than the material results
represented by these figures, greater perhaps than
the enormous relief of pain and suffering which these
sums of money have effected, are the civilising in-
fluence, the spiritual and moral benefits which have
of necessity accrued to the givers.
As a consequence of the annual appeals put forward
by preachers of every denomination a deeper interest
is aroused towards the sick poor, and a more vivid
outline is planted in the imagination of the public of
the great work that the medical charities of London
are doing in the relief of suffering. There is a
picture in the Royal Academy this year which
represents the dead Christ bound to an altar while
thoughtless people are passing by unheeding. The
only one whose attention is arrested is a hospital
nurse ; she alone, through her long acquaintance
with suffering, can understand. The idea embodied
HOSPITALS AND THEIR SPECIAL NEEDS.
Royal Dental Hospital of London, Leicester
Square, W.C.??415 to pay outstanding balance on
new operating chairs. The hospital is much hampered
by a liability of ?30,000 entailed in the erection of the
new building.
Royal Ear Hospital.??4,000 to defray balance of debt
on new building and to furnish hospital.
Royal Free Hospital.??7,000 to provide increased
accommodation, new kitchens, operating theatres, and
out-patients' department. The proposed improvements
are strongly recommended by King Edward's Hospital
Fund.
Royal Orthopaedic Hospital.?About to amalgamate
with National Orthopedic Hospital, and incorporate by
charter. Funds for building on new premises required.
Temporary address, 55 Bolsover Street, W.
Royal London Ophthalmic Hospital.?Anxious to
commemorate its hundredth year by raising ?6,000 for
current year's expenses; ?50,000 to provide for heavy
ground rent, etc.; ? 1,500 to repay balance of loan from
Charity Commissioners.
The Hospital, June 11, 1904.
20 SPECIAL HOSPITAL SUNDAY SUPPLEMENT.
in this painting might very well be transferred to our
hospitals. There are few inhabitants of this city so
degraded that they would refuse to hold out a hand
to a brother in distress whom chance placed in the
way; but, unhappily, the majority ot' people do not
see the suffering and misery that are met with in
our hospitals and therefore they are not always so
ready to come forward with assistance as they
would be in response to a direct personal appeal. It
is to the want of full knowledge that any backward-
ness on the part of the public to contribute to the
support of the voluntary hospitals is attributable,
and it is the removal of this ignorance, and the
demonstration of new and profitable fields for humane
and kindly endeavour, that is the great and lasting
work of the Metropolitan Hospital Sunday Fund.
Meanwhile it is not without profit to consider the
concrete work of the Fund, what it actually does in
one year towards assisting the sick poor by means
of pecuniary awards to the various charitable institu-
tions. Last year the sum of .?58,231 was distributed
towards the maintenance of 153 hospitals and 56 dis-
pensaries, after careful investigation into the merits
of each individual institution. In addition to this
sum, ?3,281 was devoted to supplying surgical ap-
pliances. Realising the need that existed in London
of greater facilities for obtaining surgical appliances,
it was resolved in 1881 to devote a portion of the
annual income to this object, and accordingly an
amount limited to 5 per cent, of the total receipts
has been set aside annually for this excellent pur-
pose. Some idea of the usefulness of this branch
may be gathered from the fact that no fewer than
61,303 surgical appliances have been supplied during
the last 18 years. In the course of the last 12
months the number of appliances provided amounted
to 5,232, a total which includes 98 artificial limbs,
1,590 pairs of spectacles, and 433 trusses. We men-
tion these numbers as they assist very well to bring
home to everyone who reads them what an incal-
culable blessing to the sick and afflicted poor the
work of the Metropolitan Hospital Sunday Fund
really is, and how strong is the moral obligation
upon every citizen in London in the time of his
health to contribute such a sum as he can afford to
the help of the hospitals on the 12th of June.
KING EDWARD YII. AND HOSPITAL
SUNDAY.
Last year King Edward VII., Queen Alexandra,
and members of the Royal Family demonstrated
their sympathy and interest with the work of the
Metropolitan Hospital Sunday Fund by attending
Divine Service in St. Paul's Cathedral. This
memorable event has been portrayed by Mr.
Maurice Greiffenhagen, and his fine drawing from
life has been reproduced by the Scientific Press,
28 Southampton Street, Strand, from whom a
beautiful photogravure can be obtained. It would
prove a most suitable adornment for every hospital
board-room.
HOSPITALS AND THEIR SPECIAL NEEDS.
St. George's Hospital.?That the wealthy inhabitants in
the neighbourhood should realise that its income is far
short of the necessary expenditure.
St. John's Hospital fop Diseases of the Skin,
Leicester Square, W.C.??804 deficit on current
account. New out-patients' department an urgent
necessity. The insanitary condition of in-patients' de-
partment, Uxbridge Road, calls for immediate attention.
St. Mary's Hospital, Paddington.?? 10,000 to meet the
regular wants of the current year, and ?25,000 to com-
plete and furnish the Clarence Memorial Wing.
St. Saviour's Hospital, Osnaburgh Street, N.W.?
Deficit for 1903, ?156. Increased financial aid wanted.
Samaritan Free Hospital for Women and Children,
Marylebone Road, N.W.? Removal of debt of ?1,000,
and ?16,000 still required for new out-patients' depart-
ment, bedrooms for nurses, operating theatre, and other
necessary structural works.
Seamen's Hospital, Greenwich, S.E.??8,000 to reim-
burse the general funds for outlay on much-needed
repairs and necessary improvements; ?1,000 for elec-
tric light installation; ?5,000 to pay for out-patients'
department, mortuary, and kitchen at Branch Hospital,
Royal Albert Docks.
Tottenham Hospital, N.?To provide 45 additional beds,
a new operating theatre, and proper nurses' accom-
modation, ?18,000 is required.
University College Hospital, Gower Street, W.C.?
?1,500 to furnish new out-patients'department, children's
ward, and new north wing of hospital, and ?8,000
increased income to maintain them in working order.
Victoria Hospital for Children, Chelsea, S.W.?
?15,000 to complete the new wing. ?250 to equip
two empty wards.
West End Hospital lor Diseases of Nervous
System.?To furnish new isolation ward, provide fire-
extinguishing appliances, and procure a convalescent
home by the sea.
OTHER INSTITUTIONS AND THEIR SPECIAL NEEDS.
Association for Opal Instruction of the Deaf and
Dumb, 11 Fitzroy Square, W. ?Additional support
is much needed to continue instruction in lip-reading
and articulate speech among the deaf and so-called
dumb.
Irish Distressed Ladies Fund, 411 Oxford Street, W.
?Funds greatly needed to avoid the reduction of
pensions and grants to ladies who, owing to non-receipt
of rents on property, are in poverty, and are incapaci-
tated by illness or infirmity. Others are helped to earn
their own living.
London Orphan Asylum Watford. Office, 21 Great
St. Helens, E.C.??10,000 to complete current year's
work free from debt.
Mary Wardell Nursing Home, Stamford, Middle-
sex.??G00 to repay balance of loan raised to
renew drainage. ?400 to provide for necessary im-
provements and pay current accounts.
Maternity Charity and District Nurses' Home,.
Howard's Road, Piaistow, E.?House has been
taken for treatment of serious maternity cases, for which
?200 a year is required.
The Hospital, Junk 11, 1904.
SPECIAL HOSPITAL SUNDAY SUPPLEMENT. 21
A Year's Work in the Hospitals and Medical Charities of London, 1903.
NEWINGTON AND SOUTH DISTRICT.
Comprising Battersea, Wandsworth, Tooting, Balham, Streatham, Brixton, Lambeth, Newington, Southwark
Bermondsey, Camberwell, Greenwich, Deptford, Lewisham, Blackheath, Woolwich, &c. '
602
11
25
603
269
68
57
24
40
Rebui lding
42
32
18
32
20
11
14
18
10
30
41
No. ol
Beds
Daily
Occu-
pied.
499
7
15
442
235
57
42
23
17
30
15
14
20
12
9
7
12
7
25
31
1,519
1,519
Hospitals.
Guy's
Phillips' Memorial Homoeopathic
Miller
St Thomas's
Seamen's
Evelina, for Children
Home for Sick Children
General Lying-in
Clapham Maternity & Dispensary
Royal, Waterloo
Royal Eye
Beckenham Cottage
Blackheath Cottage
Bromley Cottage
Chislehurst, &c., Cottage
Eltham Cottage
Sidcup Cottage
Livingstone Cottage
Woolwich and Plumstead Cottage
Bolingbroke Hospital
St. John's, Lewisham
Dispensaries.
Battersea Provident
Blackfriars, Provident ...
Brixton, &c.
Camberwell Provident ...
Clapham
Deptford Medical Mission
East Dulwich Provident
Forest Hill Provident ...
Greenwich Provident
Royal South London
South Lambeth, &c.
Walworth Provident
Wandsworth Common ...
Woolwich j &c., Provident
In-
patients.
Out-
patients.
7,701
136
268
6,299
2,501
683
245
556
396
192
650
239
182
383
204
165
136
137
83
453
258
21,867
21,867
128,051
600
19,413
65,703
21,968
15,340
1,790
2,235
830
5,487
25,408
301
1,096
57
"*19
6,564
294,862
29,697
5,020
5,252
7,943
1,564
2,821
6,414
1,993
2,899
3,714
2,047
79S
1,158
2,765
368,942
Total
Expendi-
ture.
?
86,795
1,062
3,750
95,155
24,655
12,695
1,880
4,048
2,012
3,529
3,856
1,007
1,544
1,663
1,091
746
602
1,014
633
4,066
2,607
254,410
3,694
252
740
1,828
371
345
895
702
668
763
506
287
277
458
266,196
Income.
Chari-
table.
?
28,001
521
2,444
4,583
11,451
2,695
1,138
1,033
392
1,825
2,574
814
1,657
1,073
790
502
374
1,096
498
3,075
1,161
67,697
106
114
579
299
252
161
111
260
74
526
318
42
51
49
70,639
Pro-
prietary.
?
34,307
224
555
51,185
4,029
3,883
233
2,652
629
882
206
10
36
257
1
31
34
16
17
147
57
99,391
60
"'30
135
19
31
13
4
18
62
6
28
99,798
Patients'
Payments.
?
4,115
302
"'90
1,274
58
262
1,104
166
557
142
156
118
400
223
211
48
106
516
424
10,272
3.395
139
112
1.396
130
83
910
487
582
"l53
159
227
360
18,405
Total
Income.
?
66,423
1,047
2,999
55,858
16,754
6,636
1,633
3,685
2,125
2,873
3,337
966
1,849
1,448
1,191
756
619
1,160
621
3,738
1,642
177,360
3,561
253
721
1,830
401
275
1,034
751
674
588
477
229
278
410
188,842
WESTM INSTER DISTRICT.?Comprising Westminster City and Liberties.
Hospitals.
Charing Cross
King's College
Westminster
Yentnor, for Consumption
Grosvenor, for Women & Children
Hospital f or Women
Gordon, for Fistula
National, for Diseases of Heart...
Royal Westminster Ophthalmic...
Royal Orthopaedic
Royal Ear...
Royal Dental ...
St. Peter's, for Stone
St. John's, for Skin
Hospital for Diseases of Throat...
Dispensaries.
Public
St. George and St. James
St. George's, Hanover Square ...
Western
Westminster General
1,566
2,556
2,681
795
232
850
305
193
743
271
269
*556
265
791
12,073
13,939
23,404
23,270
4,103
4,444
920
2,886
11,395
979
1,984
32,466
4,302
7,958
10,980
143,030
1,892
1,774
1,827
9,037
7,288
?
55,632
22,548
20,918
15,336
2,409
6,625
2,350
2,561
2,527
2,348
816
3,235
4,137
3,532
5,039
150,013
648
604
551
1,569
777
?
10,482
11,928
6,912
5,079
1,841
4,252
1,016
1,246
2,231
1,028
315
2,813
1,671
2,022
1,150
53,986
373
581
326
335
444
12.073 164.848 154.162 56.045 15.260 19.697
2,178
3,875
3,421
2,410
95
351
63
76
665
457
"*82
302
37
396
14,408
199
460
193
?
23
38
10
4,340
596
1,256
1,448
717
' 214
525
214
2,490
2,698
4,062
18,631
27
196
721
122
?
12,683
15,841
10,343
11,829
2,532
5,859
2,527
2,039
2,896
1,699
840
3,109
4,463
4,757
5,608
87,025
572
608
522
1,516
759
Legacies
not
included
in
previous
column.
?
4,714
"20
30,445
969
7,565
600
140
500
119
279
20
100
45,471
200
45,671
91,002
?
11,642
31,316
6,919
1,718
340
"280
1,324
1,592
300-
150
50
55,63L
55.631
_ The Hospital, Junk 11. 1904.
22 SPECIAL HOSPITAL SUNDAY SUPPLEMENT.
ST. MARYLEBONE AND WEST CENTRAL DISTRICT.
Comprising St. Marylebone, St. John's Wood, Bloomsbury, Holborn, &c.
No. of
Beds.
70
50
103
110
402
97
30
252
26
29
61
52
47
196
Rebui
50
26
13
60
20
16
33
1,743
1,743 1,450
No. of
Beds
Daily
Occu-
pied.
56
37
77
74
372
94
27
191
18
18
49
45
44
185
Idinrj.
bl
17
8
59
15
10
23
1,450
Hospitals.
French
Italian
London Homoeopathic
SS. John and Elizabeth
The Middlesex
Alexandra for Children
Hospital for Incurable Children
Hospital for Sick Children
S. Monica's for Children
British Lying-in
Queen Charlotte's Lying-in
New Hospital for Women
Samaritan Free
National for the Paralysed, &c,
Hospital for Epilepsy, &c.
West End, for Epilepsy, &c.
Central London Ophthalmic
Western Ophthalmic
National Orthopaedic
Hospital for Gentlewomen
National Dental
London Throat ...
Oxygen Hospital
DISPENSARIES.
Bloomsbury Provident ...
London Medical Mission
Margaret Street, for Consumption
Portland Town
St. John's Wood Provident
St. Marylebone General
Western General
In-
patients.
844
669
1,145
220
5,074
169
35
2,403
71
435
1,448
612
528
1,066
290
365
269
246
155
*732
16,864
16,864
Out-
patients.
Total
Expendi-
ture.
5,211
13,344
23,869
48,571
333
35,*421
732
1,577
15,508
6,796
6,561
890
4,561
12,964
13,993
1,663
21,480
4,682
218,156
673
9,170
701
1,423
5,531
4,083
14,289
254,026
?
4,599
2,864
11,586
4,939
68,468
5,033
1,497
21,710
1,650
2,898
5,885
5,936
5,854
21,938
1,453
5,319
1,910
1,057
3,512
2,537
1,841
1,582
1,308
185,376
259
1,364
413
139
737
882
1,360
190,530
Income.
Chari-
table.
?
4,304
3,243
8,631
2,209
14,035
4,712
894
8,742
774
654
4,396
3,695
4,744
8,342
1,181
5,094
1,909
629
2,147
868
803
382
474
82,862
69
731
229
120
280
561
1,113
85,965
Pro-
prietary.
?
9
291
3,492
420
9,162
260
109
4,476
145
1,673
383
645
294
1,811
75
71
43
106
55
90
18
Patients'
Payments.
23,628
51
167
4
10
163
60
24,086
1,024
790
*472
494
*241
111
120
1,541
2,*635
259
516
"l3
794
1,010
1,349
1,140
768
13,307
190
269
"ll
364
178
49
14,368
Total
Income.
?
4,313
3,534
13,147
3,419
23,197
5,444
1,497
13,218
1,160
2,438
4,899
5,881
5,03 3
12,788
1,515
5,681
1,952
748
2,996
1,998
2,152
1,522
1,260
119,797
259
1,054
396
135
654
902
1,222
124,419
Legaciei
not
include"
in
previofl?
column.
?
2,391
2,027
600
18,323
200
267
4,892
250
2,809
1,290
1,767
394
50
300
" 100
36,358
25
00
448
37,531.
STRATFORD AND EAST-END DISTRICT.
Comprising Bethnal Green, Tower Hamlets, West Ham, Whitechapel, Hackney, Stepney, Limehouse, Poplar, and the East.
No. of
Beds.
No. of
Beds
Daily Hospitals.
Occu-
pied.
1,565
German
London
Mildmay Mission Hospital
Poplar
West Ham, &c
Walthamstow, &c.
City of London for Dis. of the Chest
East London for Children
St. Mary's, Plaistow, for Children
East End Mothers' Home
Canning Town Cottage
Passmore Edwards Cottage, T'lb'ry
DISPENSARIES.
All Saints, Buxton Street
Eastern
Hackney Provident
London
Queen Adelaide's...
Tower Hamlets ...
Whitechapel Provident
1,565 1,275
In-
patients.
1,665
12,460
601
1,338
1,C09
573
848
2,098
588
344
133
100
21,757
21,757
Out-
patients.
29,550
182,905
12,334
37,132
23,319
2,712
9,843
33,065
15,947
422
3,864
528
351,621
1,038
7,609
1,006
2,281
6,154
3,256
2,053
375,018
Total
Expendi-
ture.
?
11,155
180,007
3,991
8,571
5,772
3,466
11,925
10,694
3,551
2,304
1,050
1,946
244,432
349
921
301
536
589
616
797
248,541
Income.
Chari-
table.
?
6,362
156,488
2,811
8,673
4,457
1,959
9,500
8,668
3,432
1,425
440
985
205,200
298
735
95
141
486
351
80
207,386
Pro-
Patients'
prietary. Payments.
?
2,604
26,311
686
1,049
465
192
224
1,201
144
779
277
132
34,064
325
*268
222
25
34,904
?
401
1,911
130
241
130
107
207
3,127
274
223
162
709
4,495
Total
Income.
?
9,367
184,710
3,627
9,963
4,922
2,151
9,724
9,869
3,706
2,311
924
1,117
242,391
298
1,334
318
409
708
538
78<
246,785
Legacie'
not
included
in
previofl'
column.
?
7,900
27,274
110
2,161
1,621
540
352
1,084
17
41,059
250
50
41,359
The Hospital, June 11, 1904.
SPECIAL HOSPITAL SUNDAY SUPPLEMENT. 23
ISLINGTON AND NORTH-WEST DISTRICT.
Comprising Islington, Holloway, Highbury, Hampstead, Highgate, St. Pancras, Stoke Newington, Tottenham, kc.
No. of
159
35
120
53
73
191
145
14
200
28
14
28
27
48
25
20
20
1,200
1,200 818
No. of
Beds
Daily
Occu-
pied.
144
26
85
44
63
165
94
13
40
16
11
19
17
34
19
9
19
818
Great Northern Central...
Hampstead General Hospital
London Temperance
North-West London
Tottenham
University College
Mount Vernon for Consumption
Children's Home Hospital, Barnet
London Fever
Invalid Asylum
Enfield Cottage
Memorial Cottage, Mildmay
St. Saviour's Home
Friedenheim Hospital ...
Willesden Cottage
Bushey Heath Cottage ...
Santa Claus Home
DISPENSARIES.
Camden Provident
Childs' Hill, Provident ...
Hampstead Provident ...
Holloway and North Islington
Islington
St. Pancras and Northern
St. Pancras Medical Mission
Stamford Hill, &c.
In-
patients.
2,081
387
1,376
615
953
2,558
560
44
371
190
169
163
138
150
222
107
39
10,123
Out-
patients.
25,998
2,460
22,937
26,079
18,860
47,215
5,762
132
149,443
1,018
1,482
11,437
2,679
11,209
1,697
1,455
10,094
10,123 1190,514 107,455
Total
Expendi-
ture.
15,084
3,367
10,944
5,310
6,633
26,022
10,832
643
11,418
852
683
1,528
2,143
3,817
1,642
761
760
J102,439
310
341
1,089
665
901
562
244
904
Income.
Chari-
table.
?
8,579
1,725
5,284
3,779
6,491
14,387
8,571
476
7 496
402
596
405
1,031
2,300
1,511
459
673
64,165
45
40
314
218
419
23 L
210
550
66,192
Pro-
prietary.
1,331
5
1,946
187
137
3,681
51
1,937
173
30
1,040
6
141
68
96
13
10,842
25
13
11
35
16
131
29
166
11,268
PatieDts'
Payments.
Total
Income.
?
608
297
279
75
95
1,136
155
16
116
926
145
72
269
69
4,333
241
287
772
239
577
112
23
6,584
?
10,518
2,027
7,509
4,041
6,628
18,163
8,622
551
10,569
730
642
1,561
1,963
2,586
1,651
824
755
79,340
311
340
1,097
492
1.012
474
262
716
84,044
Legacies
not
included
in
previous
column.
?
2,676
510
4,746
500
10,483
700
2,560
20
35
408
22,638
300
22,938
KENSINGTON AND WEST DISTRICT.
Comprising Kensington, Paddington, Bayswater, Kilburn, Chelsea, Brompton, Fulham, Hammersmith, Chiswick,
Brentford, Acton, Ealing, &c.
18
351
281
137
318
36
50
10
46
92
50
105
135
20
12
16
31
15
16
1,739
1,739
16
269
255
125
250
*50
9
36
86
41
84
94
19
9
9
18
8
9
1,387
1,387
HOSPITALS.
Queen's Jubilee
St. George's
St. Mary's
West London
Hospital for Consumption
Belgrave, for Children ...
Cheyne, for Sick & Incurable Chldn
Kensington, for Children
Paddington Green, for Children
Victoria, for Children ...
Chelsea, for Women
Cancer
Female Lock
Banstead Surgical Home
Acton Cottage
Epsom and Ewell Cottage
Reigate and Redhill Cottage
Wimbledon Cottage
Hounslow Cottage
DISPENSARIES.
Brompton Provident
Chelsea, &c.
Chelsea Provident
Kensal Town Provident
Kilburn, Maida Vale
Kilburn Provident
Notting Hill Provident
Paddington Provident
Royal Pimlico Provident
Westbourne Provident
293
4,198
4,023
2,081
1,293
Closed.
27
170
654
1,140
774
714
641
134
132
98
285
159
166
16,982
9,744
33,850
42,311
34,549
12,924
593
4,'350
16,806
19,962
2,879
1,714
779
966
?
2,032
45,424
33,725
11,331
48,210
480
2,850
1,290
4,943
7,285
5,510
13,442
5,072
665
886
857
1,381
616
611
181,432 186.610
1,412
3,342
1,986
986
2,358
6,486
737
2,700
1,839
1,172
403
3 074
264
317
538
1,279
268
531
754
430
?
1,649
13,404
12,066
7,419
13,218
1,186
1,716
939
3,690
6,032
3,464
5,638
2,430
461
1.042
954
1.043
489
455
77,295
120
347
30
47
355
60
79
158
336
46
?
77
14,621
2,743
648
6,914
24
570
96
235
719
212
4,184
28
10
57
33
6
22
211
31,410
60
150
17
"*44
11
"'24
17
50
16.982 204,450 194.468 78 873 1 31.783 7.919 11N.57*
29S
6
369
436
989
1,634
366
51
215
268
144
52
4,828
193
182
243
1,233
168
322
425
325
?
1,726
28,025
14,809
8,067
20,132
1 210
2,584
1,041
4,294
7,187
4,665
9,^22
4,092
837
1,150
1,202
1.317
655
718
113,533
373
497
229
290
399
1,304
247
504
778
421
?
50 a
11,705
14,400
2,150
19,383.
300
459?
285.
1,286
15,388.
411
"lOO
100
50
66,517
fifi 517
Thk Hospital June 11, 1904.
24 SPECIAL HOSPITAL SUNDAY SUPPLEMENT.
CITY AND EAST CENTRAL DISTRICT.
Comprising the City, St. Luke's, Shoreditch, Finsbury, and Clerkenwell.
No. of
Beds.
Ill
165
80
114
42
40
138
45
16
751
751
No. of
Beds
Daily
Occu-
pied.
Hospitals.
97
145
60
65
26
33
98
41
13
578
578
Metropolitan
Royal Free
Royal, for Diseases of the Chest,
North-Eastern, for Children
City of London Lying-in
St. Mark's, for Fistula
Royal London Ophthalmic
City Orthopedic ...
Central London Throat and Ear
Dispensaries.
Billingsgate Medical Mission ...
City
City of London and East London
Farringdon General
Finsbury
Metropolitan
Royal General
In-
patients.
1,270
2,137
788
952
608
437
2,122
300
407
9,021
9,021
Out-
patients.
34,111
39,908
6,785
19,267
2,294
1,835
39,752
1,705
9,666
155,323
3,579
6,147
26,826
2,638
10,837
5,395
2,656
213,401
Total
Expendi-
ture.
?
14,528
14,601
7,180
8,845
4,124
4,059
13,109
2,631
2,599
Income.
Chari-
table.
71,679
939
994
?
14,395
6,557
4,518
6,876
597
1,686
10,714
2,637
791
48,771
630
911
1,909 99
594
990
845
686
333
376
459
272
78,636 1 51,851
Pro-
prietary.
?
714
1,340
177
218
4,311
694
346
13
72
7,885
88
138
171
143
367
Patients'
Payments.
?
223
14
*639
3
1,734
2,613
73
2,298
231
250
257
81
8,792 1 5,803
Total
Income.
?
15,332
7,911
4,695
7,733
4,911
2,380 ! 79
11,060 611
2,650
2,597 | ...
Legacies
not
included
in
previous
column.
?
1,250
9,365
537
*200
59,269 12,042
703
999
2,535
564
797
859
720
66,446
12,042
THE MEDICAL CHARITIES OF LONDON.?A Summary of the Work Done in 1903.
It will be seen from the following summary that the Voluntary Hospitals and Medical Charities of London, during the
twelve months ending December 31st, 1903, relieved over One million eight hundred and seventy-nine thousand patients at a
cost of ?1,239,988. The Ordinary Income only amounted to ?920,113, leaving a deficiency of ?319,875 on the year's work.
The legacies received in 1903 amounted to ?281,689, being ?8,867 less than the amount received in 1902.
No. of
Beds.
1,967
751
1,082
1,743
1,739
1,200
1,565
10,047
No. of
Beds
Daily
Occu-
pied.
|1,519
578
915
1,450
1,387
818
1,275
7,942
HOSPITALS AND DISPENSARIES,
In-
patients.
Newington and South District...
City and East Central District...
Westminster District
St. Marylebone and West Central
District ...
Kensington and West District ...
Islington & North-West District
Stratford and East-End District
21,867
9,021
12,073
16,864
16,982
10,123
21,757
108,687
Out-
patients.
368,942
213,401
164,848
254,026
204,450
190,514
375,018
1,771,199
Total
Expendi-
ture.
?
266,196
78,636
154,162
190,530
194,468
107,455
?248,541
1,239,988
Income.
Chari-
table.
Pro- ! Patients'
prietary. Payments.
Total
Income.
?
70,639
51,851
56,045
85,965
78,873
66,192
1207,386
? ?
99,798
8,792
15,260
24,086
31,783
11,268
34,904
616,951 225,891
18,405
5,803
19,697
14,368
7,919
6,584
4,495
77,271
?
188,842
66,446
91,002
124,419
118,575
84,044
246,785
920,113
Legacies
not
included
in
previous
column.
?
45,671
12,042
55,631
37,531
66,517
22,938
41,359
281,689

				

## Figures and Tables

**Figure f1:**
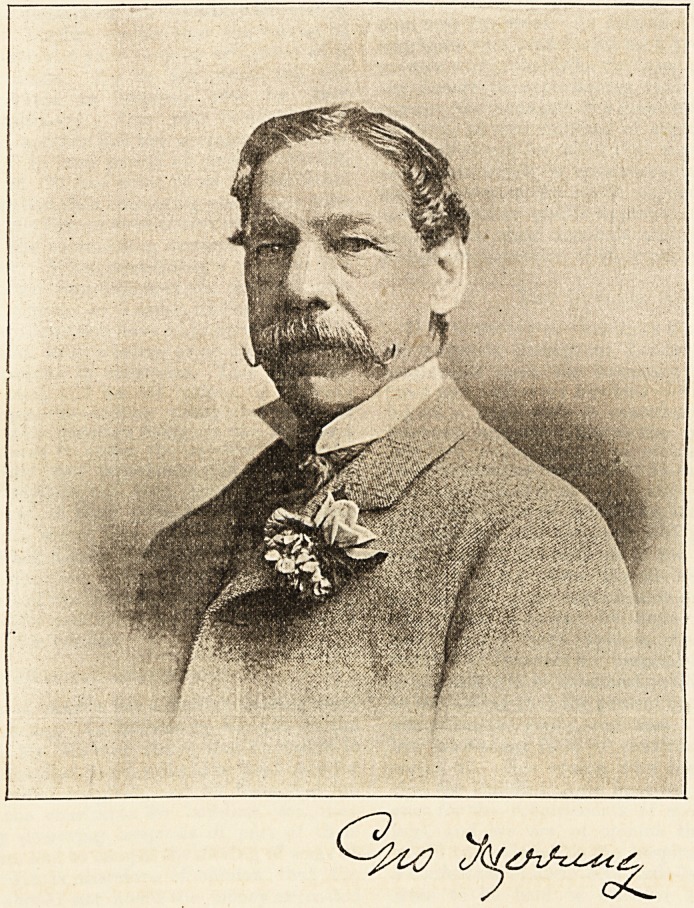


**Figure f2:**
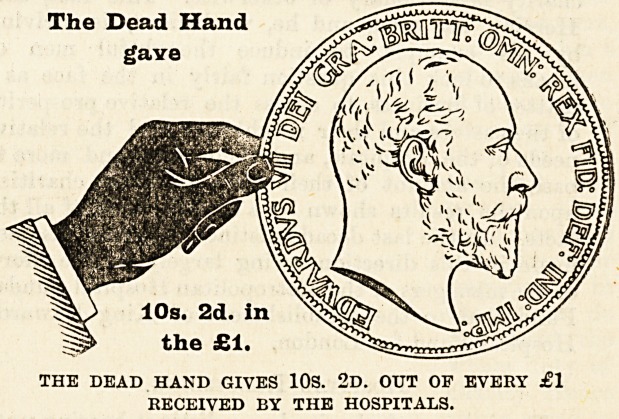


**Figure f3:**
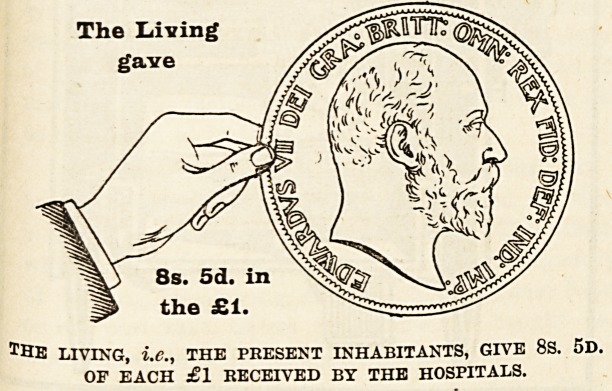


**Figure f4:**
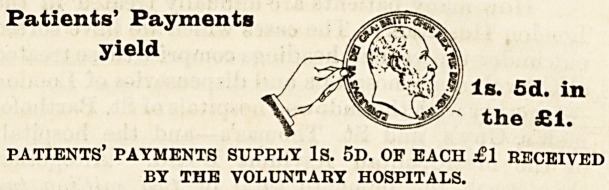


**Figure f5:**
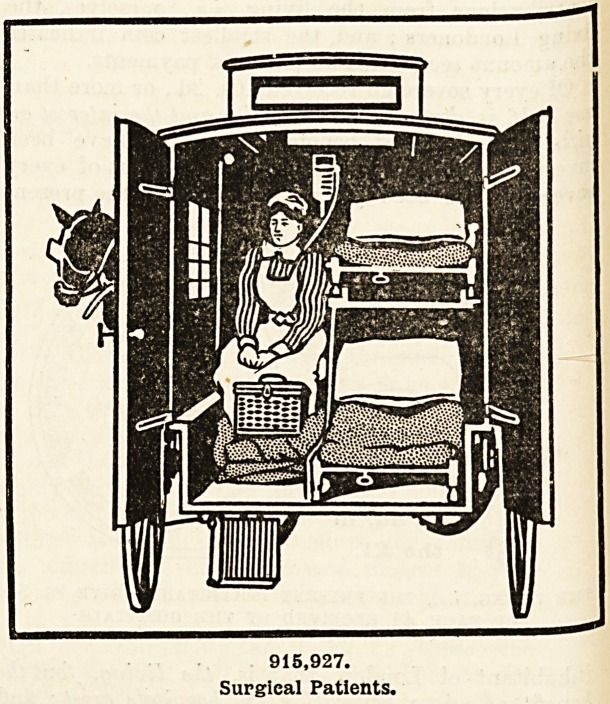


**Figure f6:**
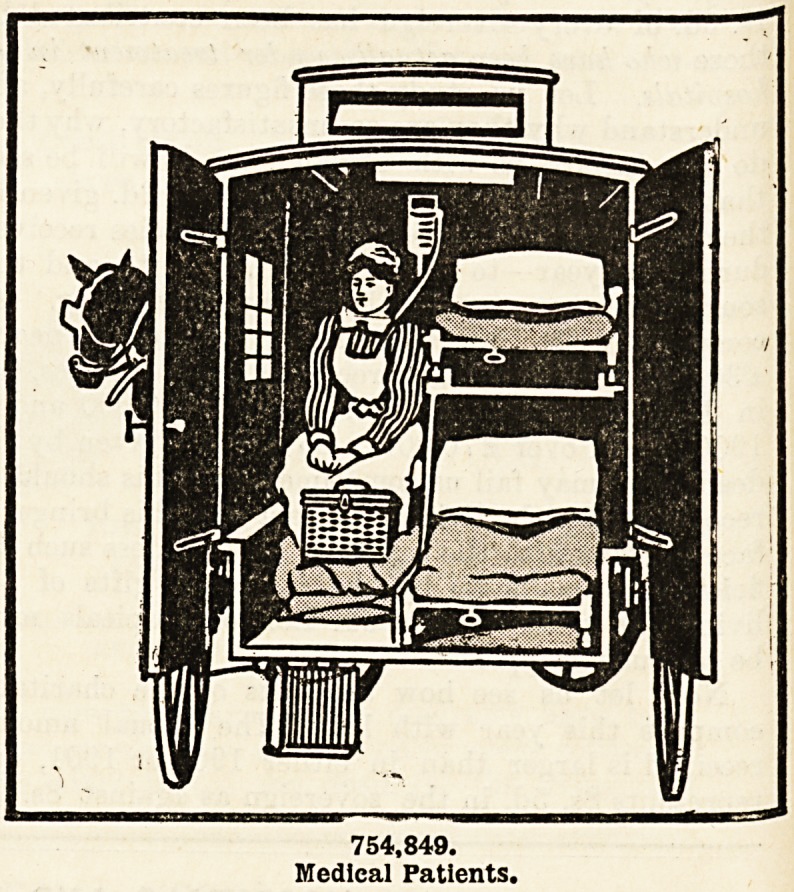


**Figure f7:**
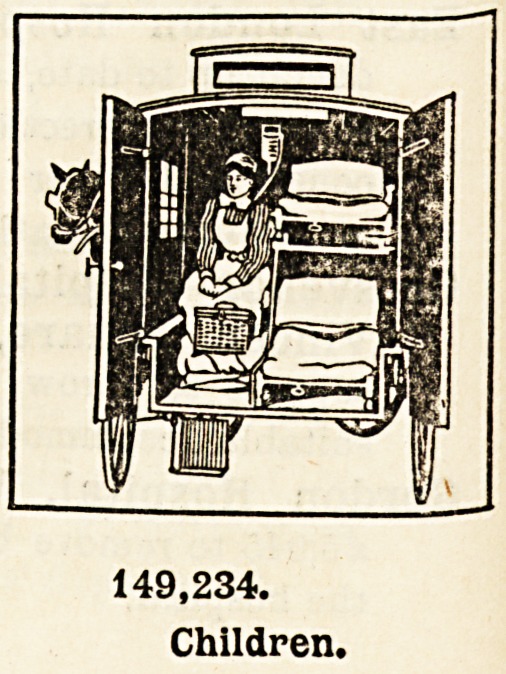


**Figure f8:**
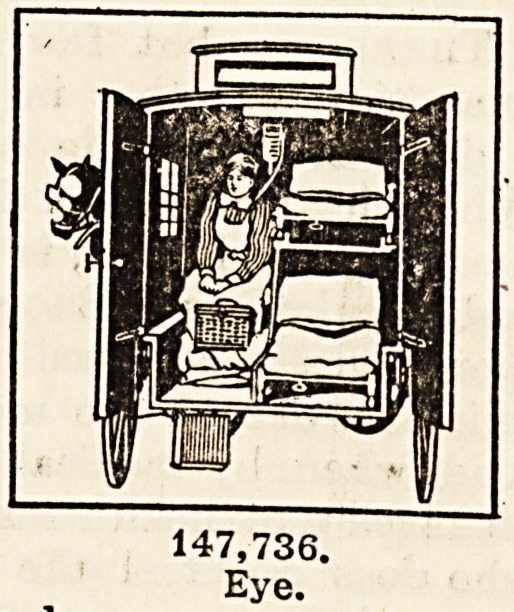


**Figure f9:**
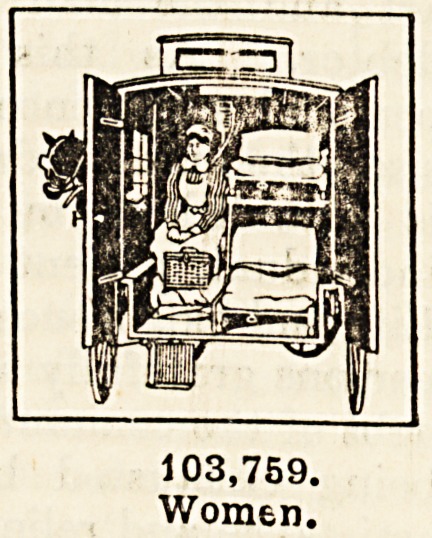


**Figure f10:**
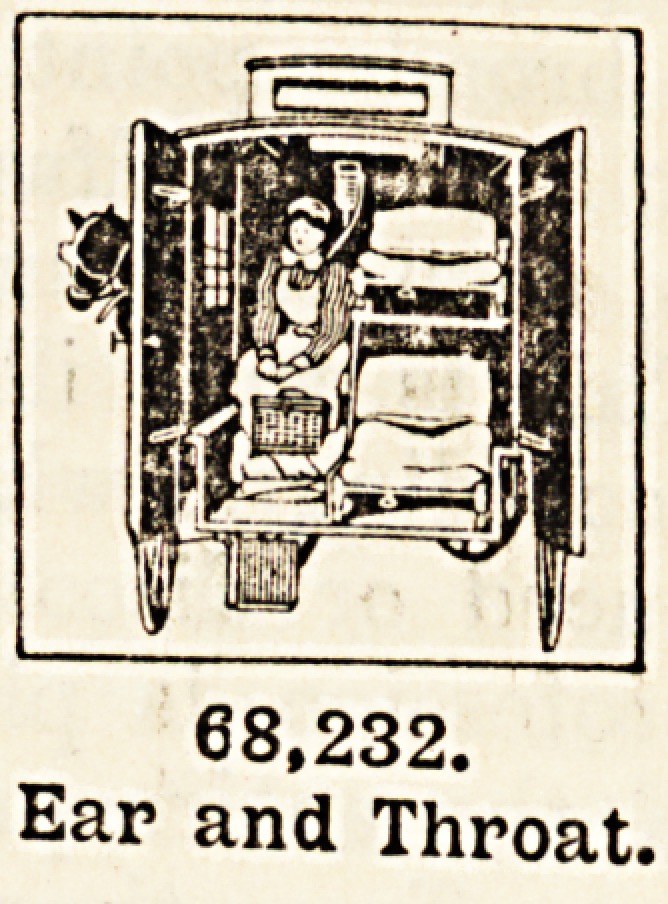


**Figure f11:**
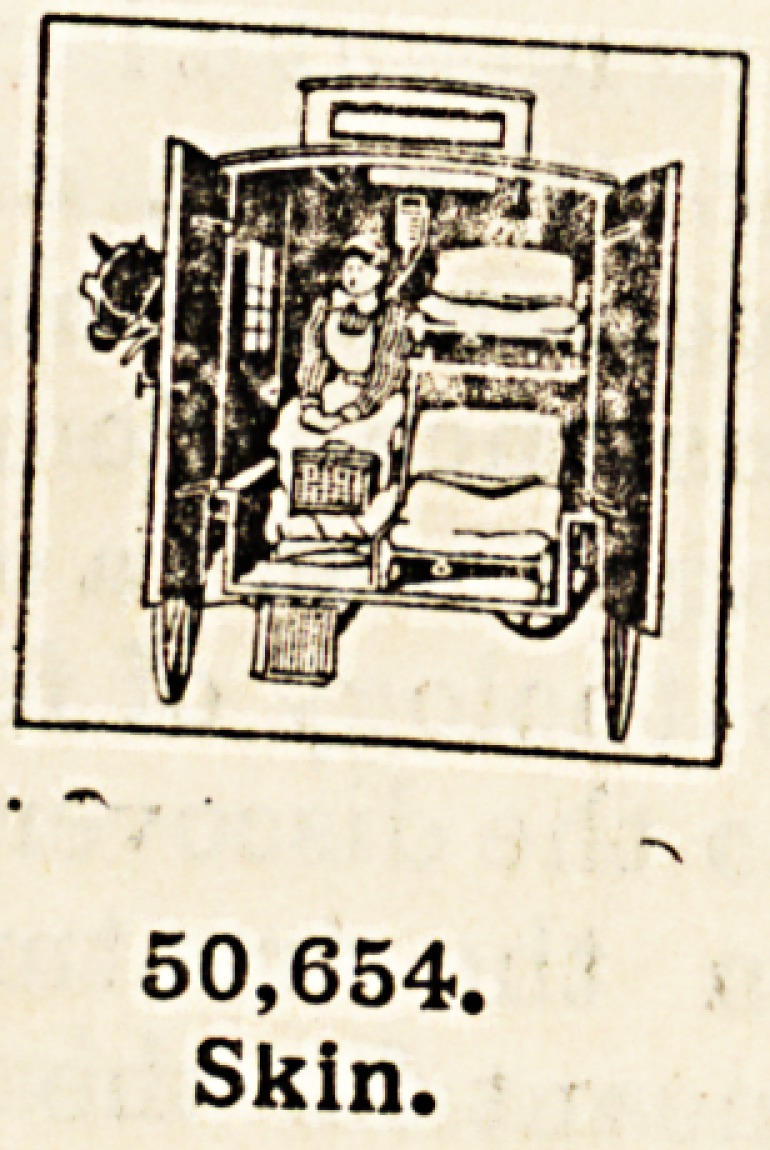


**Figure f12:**
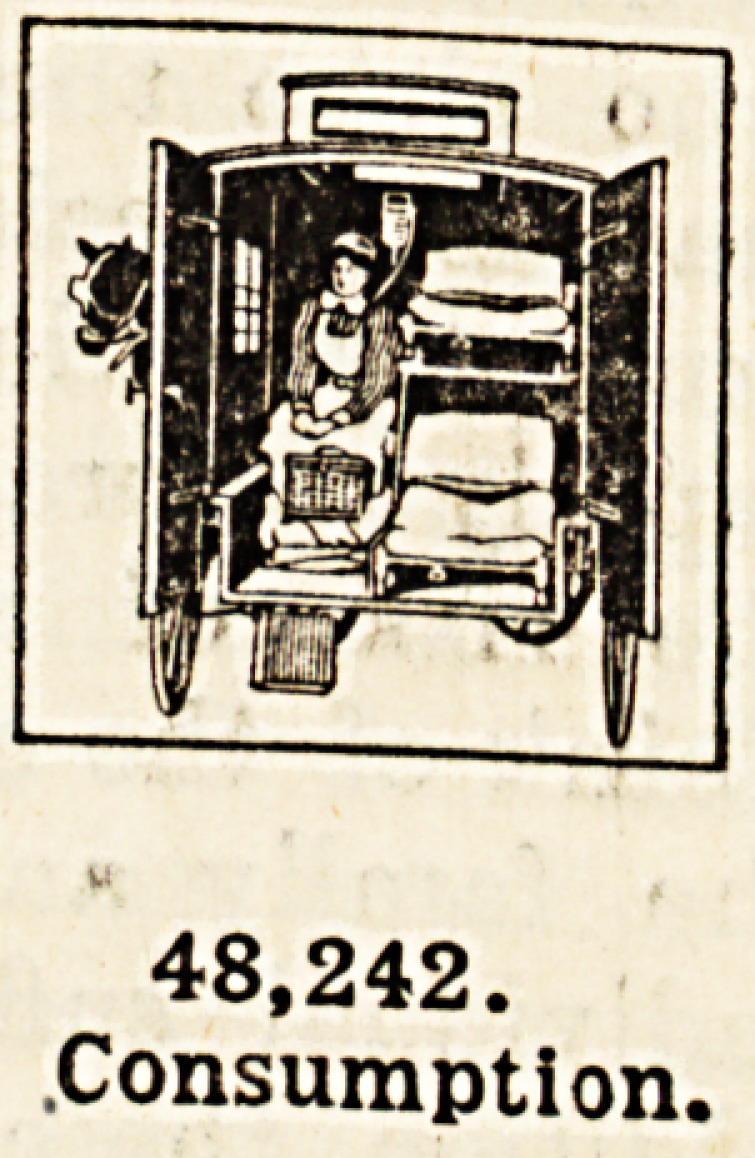


**Figure f13:**
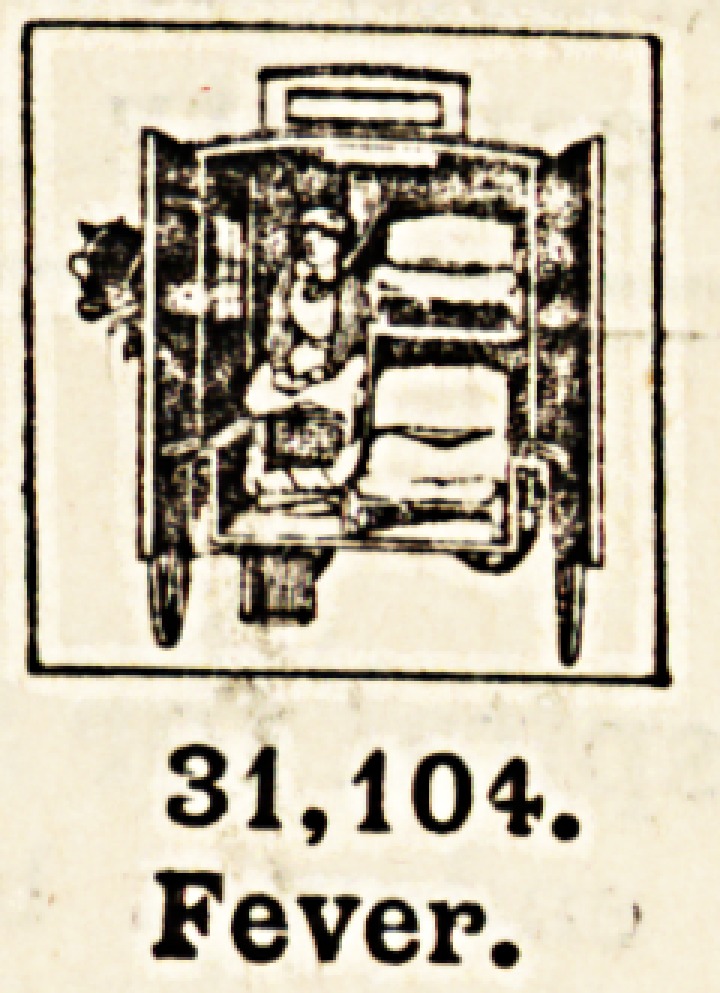


**Figure f14:**